# Family physicians’ approach to hoarseness: a qualitative study

**DOI:** 10.1017/S1463423625100704

**Published:** 2025-12-29

**Authors:** Esin Özlem Atmış, İzzet Fidancı

**Affiliations:** 1 Faculty of Health Science, Department of Audiology, Bahçeşehir University, İstanbul, Türkiye; 2 Faculty of Medicine, Department of Family Medicine, Hacettepe University, Ankara, Turkey

**Keywords:** dysphonia, family practice, hoarseness, primary care, qualitative research

## Abstract

**Aim:**

The aim of this study is to investigate family physicians’ approaches to hoarseness (dysphonia), clinical decision-making, patients’ perceptions, and structural barriers in the healthcare system using qualitative methods.

**Methods:**

Qualitative design was used. Research was reported in line with COREQ (32 items) and EQUATOR (SRQR) guidelines. Semi-structured telephone/internet interviews were conducted with 17 family physicians working primary care in Türkiye. Participants purposively sampled interviews were audio-recorded, transcribed, coded using thematic analysis, and developed themes.

**Results:**

The analysis revealed four main themes: clinical assessment and differential diagnosis, referral criteria and specialist referrals, patient perception and knowledge level, health system and structural barriers. Demographic analysis determined that veteran doctors were more sensitive to malignancy, junior doctors highlighted systemic deficits, female doctors highlighted patient behavior, while doctors who practiced in rural areas highlighted structural issues.

**Conclusion:**

Family physicians’ handling of hoarseness is not only dependent on clinical data but also on patient opinion and the health system’s conditions. For productive primary care management of hoarseness, it is recommended to (i) design guidelines and training for family physicians, (ii) increase patient education on voice hygiene and voice health, and (iii) establish health policies enhancing specialist accessibility.

## Introduction

Hoarseness (dysphonia) is a symptom that is presented to general practitioners routinely and in approximately 30% of adults at some stage of their life. Furthermore, approximately 1% of primary health care service users suggest the symptoms are frequent but not trivial (Cohen and Garrett, 2007; Bhattacharyya, 2014). For the family physician, this becomes a challenging diagnostic terrain that must be appropriately diagnosed and treated without adequate clinical instruments and direct laryngeal imaging (Schwartz *et al*., 2009).

The greatest challenges for physicians working in the context of primary health care in evaluating cases of dysphonia are that the patient does not report change in voice, presence of other conditions that appear more of a priority to patients, family physicians feeling they are not capable enough in this regard, and time constraints during the physical examination. These are constraints that hinder the diagnosis and treatment process. In addition, management programs for recurrent causes such as reflux or allergies are the most frequently used programs. Referral to an ear, nose, and throat specialist is the pathway of choice for irreversible changes in voice or speech intelligibility (Chorti *et al*., 2013).

The overall approach to evaluating hoarseness is symptomatic care and early use of conservative interventions such as voice hygiene instructions and voice therapy. Referral for specialist assessment in situations that can potentially rule out serious pathologies (e.g., laryngeal tumors) is a crucial step, however (Schwartz *et al*., 2009).

Qualitative research is a powerful tool for understanding how general practitioners perceive clinical situations and experience and reflect on managing hoarseness. Qualitative research allows us to understand cases in contextual detail and from the insiders’ perspective (Eryilmaz *et al*., 2016).

This study aims to obtain specific information on attitudes, referral decision-making, and treatment policy in practice by studying family physicians’ handling of hoarseness using qualitative methods. Furthermore, this method aims to offer insights into possible areas of education and policy development.

## Methods

### Study design and reporting standards

Reporting was conducted according to the Consolidated Criteria for Reporting Qualitative Research (COREQ) checklist (32 items) and Standards for Reporting Qualitative Research (SRQR) guidelines in the EQUATOR Network (Tong *et al*., 2007; O’Brien *et al*., 2014).

The interviews were conducted by a family phycisian veteran researcher, supported by an ENT specialist in an advisory content function. Reflexivity was emphasized to decrease the chances of bias from researchers’ clinical backgrounds, the questionnaire was pre-tested for equilibrium, and participant-centered, open research handling was utilized in the participant-researcher relationship. Thematic analysis and qualitative design were employed for study design, and data saturation was achieved with 17 family physicians with diverse backgrounds through purposive sampling. Interviews were held online/by phone, and audio recordings were anonymized and transcribed. The coding was conducted using triangulation with the assistance of family medicine and ENT specialists. Ethical committee approval and informed consent were taken from participants. For the generalizability and validity of the findings, thick descriptions (i.e., detailed contextual descriptions allowing readers to understand participants’ lives and environments in detail) were given, and non-verbal information deprivation while conducting online interviews and the lack of software support were mentioned as study limitations.

### Setting and context

The research was conducted by using family doctors working in Türkiye’s primary health care. After ethical approval, data was collected by means of online/telephone interviews (Zoom/Microsoft Teams/phone) according to the application form (Supplement 1–THE INTERVIEW FORM). Data collection was conducted by means of online and telephone interviews with family doctors working in different regions of Türkiye from July 15 to August 15, 2025, after obtaining ethical approval.

The study was organized in Ankara, but the link of invitation was distributed online through national professional networks and social media platforms for participation by family doctors from all over Türkiye. Since the survey form did not include a question regarding the participating physician’s city of practice, regional distribution could not be described. Yet, as all the participants were operating within the standardized system of Turkish primary health care, they share organizational characteristics towards service delivery, patient population, and working conditions, which are conducive to the interpretability and generalizability of the results.

### Participants and sample

Purposive sampling was used for taking part in the study. Inclusion criteria were (i) active practice in family medicine in Türkiye, (ii) ≥1-year professional experience, (iii) 18–65 years old, and (iv) informed consent. Exclusion criteria were working outside primary care, not a specialist in family medicine, having serious communication difficulties, and having active serious psychiatric disorder.

The sample was set by using purposive sampling; data collection continued until thematic saturation. Although the ceiling in the application form was set as 25 participants, in field application reported in the present article, interviews were conducted with *n* = 17 family physicians, and thematic saturation was achieved. Participant diversity (age, gender, years of experience) was assured; participants were anonymized with the codes FP-1. FP-17.

Purposive sampling design was constructed to allow for maximum variation in gender, years of professional work, and practice site (urban/rural). Potential informants were familiar through professional associations and invited using institutional email. Physicians from different regions and socio-economic groups were purposively sampled to gain access to the range of primary care locations in Türkiye. Recruitment was stopped after 17 interviews once data saturation was reached and no new themes or codes were observed.

### Data collection

A semi-structured interview schedule was employed to gather data. The interview schedule was formulated following a review of literature and consultation with an ear, nose, and throat specialist. The questions were designed to discuss physicians’ handling of hoarseness, diagnosis, referral, voice hygiene, and patient education attitudes.

The researcher conducted interviews online (Microsoft Teams, Zoom) or over the phone, and all lasted approximately 20–30 minutes (average interview duration: 24.7 minutes). By the participants’ consent, audio recordings were made, interviews were transcribed within 24 hours, and the original audio recordings were securely deleted.

Data were collected with a semi-structured interview tool developed from literature and clinical expert consensus (DiCicco-Bloom and Crabtree, 2006; Gill *et al*., 2008; Kallio *et al*., 2016). The tool covered history taking for hoarseness (duration, risk factors, concomitant symptoms), differential diagnosis, referral decisions, voice hygiene teaching, and systemic barriers. Phone or internet interviews, at the participant’s choice, were audio recorded (with participant agreement).

### Data management and privacy

Audio files were transcribed and erased within 24 hours; the original files were then securely and permanently deleted. Transcripts were anonymized (codes only were used; no identifying details such as names or institutions were collected). Data were stored in encrypted storage facilities, and only authorized members of the research team could access them. The entire process was conducted in accordance with the principles of the Helsinki Declaration and the Law on Protection of Personal Data; subjects were given the option of withdrawal from the study at any time and destruction of their data.

### Analysis

Text data were analyzed using thematic analysis (11). Open coding was performed first; minor themes were derived from the codes, followed by major themes. The first round of coding was performed by a family medicine specialist; the codes/themes and analytical decisions were discussed with an external expert (ear, nose, and throat diseases specialist) and finalized through inter-researcher consensus (analytical triangulation). Software was not used; manual analysis was performed. The findings were enriched with direct quotations, and thick description was provided to ensure consistency between themes (Van Houtte *et al*., 2011).

### Ethics

Ethical approval for the study was obtained from the Acıbadem Mehmet Ali Aydınlar University Medical Research Evaluation Board with the number ATADEK-2025/12 (Date: 10.07.2025; Decision no: 2025-12/101). Informed consent was posted online; confidentiality and voluntariness were ensured. No incentives/rewards were given in the study; there is no conflict of interest.

## Limitations and contributions

Because the study was conducted in a single nation and among a single occupational group, its context sensitivity is intrinsic to transferability. Online/telephone interviewing has the potential for restricted pickup of nonverbal signals. However, the dependability of findings was enhanced through triangulation among experts, thick description, and closure based on saturation.

## Results

**Figure 1.**
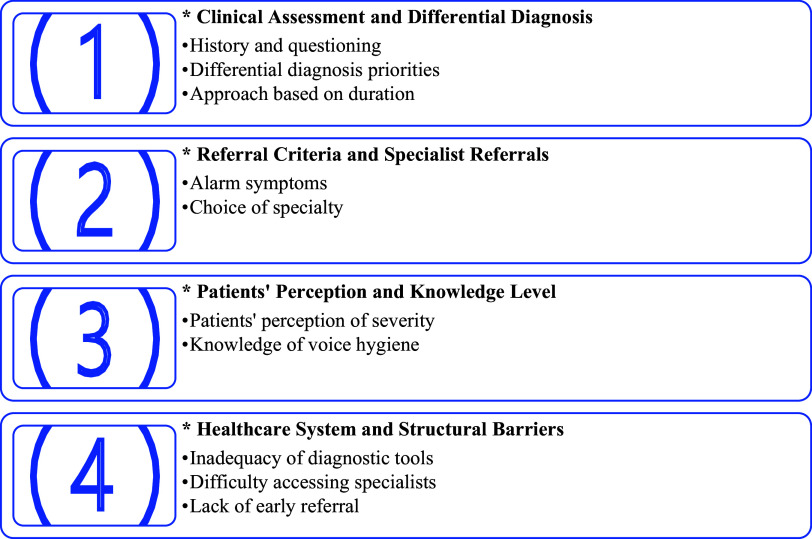
Main themes identified in the thematic analysis. *: Main theme; **·**: Minor theme.

The experiences and approaches of 17 family physicians participating in the study regarding hoarseness were analyzed. Based on the coding, four main themes and related minor themes emerged: (1) Clinical assessment and differential diagnosis, (2) Referral criteria and specialist referrals, (3) Patients’ perceptions and level of knowledge, (4) Health system and structural barriers. Each theme is presented below, supported by direct quotations from participants’ statements (Figure 1).

As illustrated in Table 1, participant demographics such as age, gender, years in practice, and workplace context were closely associated with the thematic emphases identified during the analysis, highlighting differences in clinical sensitivity, patient perception, and recognition of systemic barriers.

**Table 1.** Participant demographic characteristics and thematic emphases

Participant	Age	Gender	Years in profession	Thematic highlights
FP-1	41	Female	15	Duration >2 weeks → referral; lack of diagnostic tools at the Family Health Center
FP-2	34	Male	9	Sensitive to malignancy and alarm symptoms; referral difficulties in rural areas
FP-3	32	Female	7	Neck mass → immediate referral; lack of knowledge about voice hygiene
FP-4	40	Male	16	Considers duration critical in medical history; emphasizes that patients consider it insignificant
FP-5	41	Female	15	Early referral of professional voice users; frequently reminds of malignancy
FP-6	31	Male	6	Explains acute cases with viral causes; complaints of systemic obstacles
FP-7	31	Male	7	Rapid referral for hemoptysis/weight loss; prioritizes alarm symptoms
FP-8	35	Male	9	Emphasizes that patients present late and have problems with the central appointment system
FP-9	28	Female	4	Sensitive to risk factors (smoking, age) despite being young
FP-10	29	Male	5	Reports that patients downplay their complaints; late presentation is common
FP-11	44	Female	15	Smokers perceive hoarseness as normal
FP-12	32	Female	7	Considers sudden deterioration in voice quality critical; refers early
FP-13	29	Female	4	Emphasizes that patients may be unaware that reflux can cause hoarseness
FP-14	38	Male	10	Considers occupational voice use (teachers, imams) as high risk; stresses early referral for persistent hoarseness
FP-15	45	Female	18	Highlights patient reluctance to stop smoking; links persistent hoarseness to lifestyle and psychosocial factors
FP-16	33	Female	6	Sensitive to gastroesophageal reflux as a cause; emphasizes diet and lifestyle counseling along with referral
FP-17	50	Male	20	Stresses importance of laryngoscopy at secondary care; complains about limited access to ENT specialists in rural areas

FP: Family Physician.

### Theme 1. Clinical evaluation and differential diagnosis

Most physicians stated that when patients present with hoarseness, they first inquire about the duration of the complaint, accompanying symptoms, and risk factors.

- *‘If hoarseness lasts longer than two weeks, I take a detailed history and specifically inquire about smoking and occupational factors.’* (FP-1)

Duration was viewed as a key diagnostic clue.

- *‘My first question is usually ‘How long has it been going on?’ Because the duration tells me a lot.’* (FP-4)

Physicians considered infectious causes to be the most likely in acute cases, while considering the possibility of malignancy in prolonged ones.

- *‘We frequently encounter acute upper respiratory tract infections, so if it’s short-term, I usually attribute it to viral causes.’* (FP-6)
- *‘If hoarseness persists for more than three weeks, I always consider the possibility of malignancy.’* (FP-5)
- *‘I evaluate hoarseness more carefully in patients who smoke and are over 40 years old.’* (FP-9)

### Theme 2. Referral criteria and specialist referrals

Participants stated that, in addition to the duration of hoarseness, they referred patients to an ENT specialist without delay when they observed alarm symptoms (such as hemoptysis, neck mass, weight loss).

- *‘If there is a palpable mass in the neck, I immediately consider the possibility of malignancy.’* (FP-3)
- *‘If there is hemoptysis or weight loss, I refer them to ENT without delay.’* (FP-7)

Physicians also reported that any persistent hoarseness beyond two weeks was a clear reason for referral.

- *‘Any hoarseness lasting longer than two weeks is a referral reason for me.’* (FP-2)

Some participants emphasized early referral for professional voice users, including teachers and imams.

- *‘I refer individuals who use their voice professionally to ENT, even if it’s just for a short period.’* (FP-5)

Rapid deterioration in voice quality was also considered an urgent indicator for referral.

- *‘For me, the most critical point is observing a rapid deterioration in the patient’s speech quality.’* (FP-12)

### Theme 3. Patients’ perception and knowledge level

Physicians reported that most patients do not pay attention to short-term hoarseness in the early stages but seek medical attention when the condition persists.

- *‘Most patients neglect it, saying ‘my voice has always been like this’.’* (FP-4)

They noted that patients typically seek medical care only when the symptom persists.

- *‘They start to worry when it persists, but they generally don’t see a doctor in the early days.’* (FP-8)

Some physicians explained that smokers often normalize hoarseness as a usual condition.

- *‘Many smokers perceive hoarseness as a normal condition.’* (FP-11)

Physicians also acknowledged their own lack of confidence in providing voice hygiene education.

- *‘I try to give voice hygiene recommendations, but I don’t feel very confident myself.’* (FP-3)

Awareness of reflux-related voice problems was limited among patients.

- *‘Most patients are unaware that hoarseness can be caused by reflux.’* (FP-13)

### Theme 4. Health system and structural barriers

Participants frequently mentioned the lack of diagnostic tools in family health centers as a significant limitation.

- *‘We have no means to perform a larynx examination at the Family Health Center.’* (FP-1)

Difficulties in obtaining ENT appointments through the central appointment system were also emphasized.

- *‘Sometimes patients we refer to ENT cannot find an appointment for weeks.’* (FP-8)

Physicians noted that patients often delay seeking care, which contributes to late diagnoses.

- *‘Patients often do not take their complaints seriously and delay seeking care.’* (FP-10)

Those working in rural areas described additional logistical challenges related to referrals.

- *‘Since I work in a rural area, even the transportation of referred patients becomes an issue.’* (FP-2)

Several participants stressed that these systemic barriers limit early diagnosis and timely management.

- *‘Due to systemic barriers, early diagnosis is often not possible.’* (FP-6)

### Relationship between participant demographics and themes

Participants’ ages ranged from 28 to 50 (mean: 36.1 years), and their professional experience ranged from 4 to 20 years. The analysis revealed some differences between demographic characteristics and approaches to hoarseness.

### Findings related to demographic characteristics

- Experienced physicians (≥15 years): More frequently raised suspicion of malignancy (e.g., FP-1, FP-4, FP-5, FP-11).
- Young physicians (<5 years of experience): Focused more on risk factors (smoking, age) and emphasized systemic deficiencies (e.g., FP-9, FP-13).
- Female physicians: They drew more attention to patients’ perceptions and neglectful behaviors (e.g., FP-4, FP-11, FP-13).
- Physicians working in rural areas (e.g., FP-2): More clearly expressed difficulties with transportation and appointments after referral.
- Those who encountered professional voice users (e.g., FP-5): This group mentioned early referral decisions more frequently.

### Comprehensive interpretation of themes

Findings of this study reveal that the management of hoarseness among family physicians is complex. In the clinical evaluation process, physicians prioritize duration and risk factors in differential diagnosis, while they place special value on alarm symptoms in referral criteria. However, uncertainty and information gaps in patients’ knowledge directly affect the diagnosis and treatment processes. The narratives of the interviewees indicate that both physicians and patients need to be informed about voice hygiene.

The structural barriers and the health system become one of the most pronounced limiting factors in these clinical processes. Insufficient diagnostic equipment at primary care, difficulties in obtaining appointments through the central appointment system, and access problems after referral in rural settings disrupt early diagnosis and effective referral processes.

Demographically, it is noteworthy that older physicians are more sensitive to malignancy, while younger physicians concentrate on systemic concerns and risk factors. Female physicians’ observations regarding patient behavior take precedence, while physicians working in rural settings elaborate more on healthcare system barriers.

When all these findings are considered, the treatment of hoarseness among general practitioners is strongly influenced not only by clinical knowledge and experience but also by patient perception, educational level, and health system conditions. Such a multifaceted framework implies that education programs, clinical practice guidelines, and system improvements must be addressed at the same time if hoarseness is to be more effectively treated at the primary care level.

## Discussion

This study examined family physicians’ approach to hoarseness from a qualitative perspective and revealed that clinical decision-making processes have a multidimensional structure. The findings highlight the critical role of time in clinical assessment and differential diagnosis, the prominence of alarm symptoms in referral decisions, patients’ low awareness of hoarseness, and the challenges posed by healthcare system barriers to management processes.

### Clinical assessment and referral decisions

Most participants stated that they considered the possibility of malignancy in cases of hoarseness lasting longer than two to three weeks. This finding is consistent with the American Academy of Otolaryngology-Head and Neck Surgery’s guidelines on hoarseness (dysphonia), which recommend that patients with hoarseness lasting longer than 4 weeks be referred to an ENT specialist for laryngeal evaluation (Schwartz *et al*., 2009). Previous studies have also shown that duration is the most significant determinant in family physicians’ approach to hoarseness (Cohen and Garrett, 2007). However, our study revealed that, in addition to duration, participants made earlier referral decisions, particularly for professional voice users (teachers, imams, etc.). This finding is consistent with studies in the literature reporting that voice disorders in professional voice users lead to significant quality of life losses in the early stages (Van Houtte *et al*., 2011).

### Patients’ perception and knowledge of voice hygiene

According to participants’ accounts, patients tend to dismiss short-term hoarseness as insignificant. This aligns with the low awareness and delayed presentation rates reported in Bhattacharyya’s large-scale study of US adults (Bhattacharyya, 2014). Additionally, our study highlighted a lack of knowledge regarding voice hygiene education among both physicians and patients. The literature also emphasizes that lack of awareness about voice hygiene complicates both diagnosis and compliance with conservative treatment (Behrman *et al*., 2008). This finding highlights the importance of programs targeting patient and physician education in primary care.

### Health system and structural barriers

Participants frequently mentioned the lack of diagnostic tools in family health centers, as well as difficulties in finding ENT appointments through the center appointment system and access problems in rural areas. This situation is like the structural barriers frequently reported in primary health care in Türkiye (Chorti *et al*., 2013; Eryilmaz *et al*., 2016). Furthermore, previous studies have also indicated that healthcare system-related barriers play a significant role in delays in early referral processes (Cohen and Garrett, 2007). Our findings suggest that, without systemic reforms, the clinical awareness of family physicians alone may not be sufficient to ensure early diagnosis.

### Experience differences

Our study found that experienced physicians (>15 years) were more sensitive to malignancy, while younger physicians emphasized systemic deficiencies more. Similarly, it was noted that female physicians focused on patient perception and behavior, while those working in rural areas highlighted transportation and appointment issues. These differences are explained in qualitative literature by the concept of ‘clinical reflexivity’; it is stated that physicians’ experience, gender, and work context are decisive in patient management (Nowel *et al*., 2017).

### Contribution and recommendations of the study

This research shows that hoarseness in primary care is shaped not only by clinical knowledge but also by patient perception, educational gaps, and healthcare system barriers. The findings emphasize the need to develop training programs for family physicians that cover voice hygiene, risk factors, and referral criteria for. Additionally, improvements to the central appointment system and the development of policies facilitating access to specialists in rural areas could strengthen early diagnosis and treatment.

### Limitations

The study was conducted only in Türkiye with 17 participants; therefore, the generalizability of the findings is limited. Furthermore, due to online/telephone interviews, non-verbal communication data were limited. Therefore, thematic saturation, participant diversity, and direct quotations were used to enhance the reliability of the analysis.

## Conclusion

This study validated that clinical evidence alone does not dictate the practice patterns of family physicians for hoarseness but is influenced by patient attitudes and organizational factors in healthcare. To effectively address hoarseness in primary care, there must be a boost in physician training, voice hygiene education among patients, and advanced referral systems. Policy and educational interventions must be implemented in future research to equip family physicians with early diagnosis and management of hoarseness.

## Supporting information

Atmış and Fidancı supplementary material Atmış and Fidancı supplementary material

## Data Availability

No new data were generated or analyzed in support of this research.

## Supplementary material

The supplementary material for this article can be found at https://doi.org/10.1017/S1463423625100704.
