# Supplementary material for: Family physicians’ approach to hoarseness: a qualitative study
Source: Prim Health Care Res Dev. 2025 Dec 29;27:e4. doi: 10.1017/S1463423625100704 (PMC12867484; doi:10.1017/S1463423625100704)
Supplement: Atmış and Fidancı supplementary material [file S1463423625100704sup001.pdf]

# THE INTERVIEW FORM

DEMOGRAPHIC QUESTIONS

1) Age:

2) Gender:

3) Marital status:

4) Do you have children?

4) How many years have you been in your profession?

OPEN-ENDED QUESTIONS

1. When you encounter a patient presenting with hoarseness, what do you first inquire about and what do you pay attention to?

2. In a patient presenting hoarseness, what conditions do you primarily consider in terms of differential diagnosis?

3. How does the duration of hoarseness (e.g., a few days vs. more than two weeks) affect your approach?

4. What tests or assessments, other than a physical examination, do you perform or refer to a patient presenting with hoarseness to assist in diagnosis?

5. When you suspect a serious condition (e.g., malignancy, vocal cord paralysis) in hoarseness, how do you recognize this? Which symptoms alarm you?

6. When evaluating the need for referral in patients presenting with hoarseness, what criteria do you use to make your decision?

7. To which department do you usually refer such patients? (ENT, chest diseases, etc.) What factors are decisive in your referral decision?

8. In your opinion, how seriously do patients presenting with hoarseness usually take this condition? If there is a finding that concerns you, how do you explain it to the patient?

9. Do you think patients are sufficiently knowledgeable about voice hygiene or voice protection? What kind of recommendations do you make when you think it is necessary?

10. In your opinion, what is the biggest shortcoming in the system regarding the diagnosis and treatment of patients presenting with hoarseness? What kind of obstacles do you encounter that make diagnosis or referral difficult?
